# Effect of Adjuvant Radiation Dose on Survival in Patients with Esophageal Squamous Cell Carcinoma

**DOI:** 10.3390/cancers14235879

**Published:** 2022-11-29

**Authors:** Weiming Han, Xiao Chang, Wencheng Zhang, Jingsong Yang, Shufei Yu, Wei Deng, Wenjie Ni, Zongmei Zhou, Dongfu Chen, Qinfu Feng, Jun Liang, Zhouguang Hui, Lvhua Wang, Shugeng Gao, Yu Lin, Xiaohui Chen, Junqiang Chen, Zefen Xiao

**Affiliations:** 1 Department of Radiation Oncology, National Cancer Center/National Clinical Research Center for Cancer/Cancer Hospital, Chinese Academy of Medical Sciences and Peking Union Medical College, Beijing 100021, China; 2 Key Laboratory of Carcinogenesis and Translational Research (Ministry of Education/Beijing), Department of Radiation Oncology, Peking University Cancer Hospital & Institute, Beijing 100142, China; 3 Department of Radiation Oncology, Tianjin Medical University Cancer Institute and Hospital, National Clinical Research Center for Cancer, Tianjin 300060, China; 4 Department of Radiation Oncology, Union Hospital, Tongji Medical College of Huazhong University of Science and Technology, Wuhan 430030, China; 5 Department of Radiation Oncology, Beijing Chaoyang Hospital, Capital Medical University, Beijing 100020, China; 6 Department of Radiation Oncology, Beijing Shijitan Hospital, Capital Medical University, Ninth School of Clinical Medicine, Beijing 100038, China; 7 Department of Thoracic Surgery, National Cancer Center/National Clinical Research Center for Cancer/Cancer Hospital, Chinese Academy of Medical Sciences and Peking Union Medical College, Beijing 100021, China; 8 Department of Radiation Oncology, Fujian Medical University Cancer Hospital, Fujian Cancer Hospital, Fuzhou 350014, China; 9 Department of Thoracic Surgery, Fujian Medical University Cancer Hospital, Fujian Cancer Hospital, Fuzhou 350014, China

**Keywords:** esophageal cancer, adjuvant radiotherapy, adjuvant radiation dose, local-regional recurrence-free survival, overall survival

## Abstract

**Simple Summary:**

For patients with esophageal cancer, postoperative radiotherapy (PORT) improved LRFS. However, concerns about treatment-related toxicity issues limited its application. The authors found that for patients receiving upfront surgical resection, with adjuvant radiation dose (aRTD) escalation, the hazard ratio (HR) of LRFS declined until aRTD exceeded 50 Gy, then remained steady. However, HR of treatment-related mortality was stable until aRTD exceeded 50 Gy, then it increased. There is an adequate aRTD that can afford balanced PORT-related LRFS enhancement and related toxicity. The authors highlighted that clinicians should be aware that PORT has the potential to improve unfavorable LRFS and survival outcomes in ESCC patients treated with upfront surgery. The findings of the current study could serve as evidence for delivering appropriate aRTD and designing additional prospective stratified randomized controlled trials.

**Abstract:**

**Background:** For patients with esophageal squamous cell carcinoma (ESCC) treated with surgery alone, the incidence of local-regional recurrence remains unfavorable. Postoperative radiotherapy (PORT) has been associated with increased local-regional recurrence-free survival (LRFS), although its application is limited by concerns of PORT-related toxicities. **Methods:** Among 3591 patients with ESCC analyzed in this study, 2765 patients with T3-4N0 and T1-4N1-3 lesions and specific local-regional status information were analyzed in a subsequent analysis of adjuvant radiation dose (aRTD) effect. Application of the restricted cubic spline regression model revealed a non-linear relationship between aRTD and survival/radiotoxicity. Linear regression analysis (LRA) was performed to evaluate correlations between LRFS and overall survival (OS)/ disease-free survival (DFS). **Results:** For patients staged T1–2N0, T1–2N1–3, T3–4N0, and T3–4N1–3, 5-year OS in PORT and non-PORT groups were 77.38% vs. 72.91%, *p* = 0.919, 52.35% vs. 46.60%, *p* = 0.032, 73.41% vs. 61.19%, *p* = 0.005 and 38.30% vs. 25.97%, *p* < 0.001. With aRTD escalation, hazard ratios (HRs) of OS/DFS declined until aRTD exceeded 50Gy, then increased, whereas that of LRFS declined until aRTD exceeded 50 Gy, then remained steady. HR of treatment-related mortality was stable until aRTD exceeded 50 Gy, then increased. LRA revealed strong correlations between LRFS and OS/DFS (r = 0.984 and r = 0.952, respectively). An absolute 1% advancement in LRFS resulted in 0.32% and 0.34% improvements in OS and DFS. Conclusions: An aRTD of 50Gy was well-tolerated, with favorable survival resulting from PORT-related LRFS improvement in patients staged T3–4N0 or T1-4N1–3. Further stratification analyses based on tumor burden would help determine potential PORT-beneficiaries.

## 1. Introduction

Surgery is one of the most important curative approaches for esophageal cancer. The CROSS [1] and NEOCRTEC5010 [2] trials revealed that neoadjuvant chemoradiotherapy followed by surgery could afford survival benefits. Nevertheless, the FFCD 9901 [3] trial failed to reveal superior survival in patients treated with neoadjuvant chemoradiotherapy comparing with those who underwent surgery alone among those with the American Joint Committee on Cancer Tumor-Node-Metastasis stage I and II. The prognosis was varied even among patients treated with similar approaches. Therefore, patients must be divided into several strata for appropriate treatment. Over 50% of patients with esophageal cancer receive surgical resection as primary management in real-world clinical practice of cancer because of patients’ or doctors’ preference for achieving early dysphagia relief through upfront surgical resection, concerns of neoadjuvant therapy-related toxicity, and the advantages that an accurate pathologic stage can be determined from the initial surgery and unnecessary neoadjuvant therapy can be avoided in patients with early-stage disease [4,5]. For patients treated with surgical resection without adjuvant therapy, the probability of local-regional recurrence ranged from 23.0% to 56.5%, accounting for 55.6–84.5% of disease recurrence [6,7,8,9,10]. Once the disease has recurred, the subsequent prognosis could be dismal. The median survival time after postoperative disease recurrence ranged from 3 to 8 months [10,11,12]. 

Postoperative radiotherapy (PORT) remains one of the potential treatment approaches for delaying local-regional recurrence, achieving superior disease-free survival (DFS), or overall survival (OS) in select patients [13,14,15,16,17,18,19,20,21]. It is essential to identify patients who may benefit from PORT. In the majority of previous studies on PORT, an adjuvant radiation dose (aRTD) of 50–60 Gy was used [14,15,22,23,24,25,26,27]. However, few studies have evaluated the impact of aRTD on survival outcomes and treatment-related toxicities in patients receiving PORT in the era of intensity-modulated radiotherapy (IMRT) [28,29]. Furthermore, concerns about PORT-related toxicities, which might lead to a low quality of life (QoL) due to persistent anastomotic stenosis-related dysphagia or treatment-related mortality, limit the application of PORT. Moreover, whether an improvement in PORT-related local-regional recurrence-free survival (LRFS) could translate into improved OS or DFS remains unclear. 

The current study aimed at exploring the effect of aRTD on survival outcomes and treatment-related toxicity, identifying the adequate aRTD that could afford favorable survival outcomes and acceptable toxicities, and evaluating the correlation between LRFS enhancement and DFS, OS improvements in patients treated with radical surgical resection through the data from two cancer centers in China. As the pathology in 95.5% of Chinese patients was squamous cell carcinoma [30] and there were distinctive differences in molecular features which might affect the categorization of prognosis comparing with adenocarcinoma [31], only patients pathologically diagnosed with esophageal squamous cell carcinoma (ESCC) were enrolled in the current study.

## 2. Methods

### 2.1. Patient Population

This retrospective study was performed at two cancer centers in China and approved by the cancer centers’ ethics committee. Between January 1993 and December 2012, 3811 patients diagnosed with esophageal carcinoma after surgery were under evaluation. To identify those who may benefit from PORT, 3591 patients were available for analysis in the current study after excluding the following individuals: those diagnosed with other malignancies within 5 years before surgery (n = 62), recurrence or port-site tumor implantation and undergoing palliative-intended radiotherapy (n = 19), or adenosquamous carcinoma or basal cell-like carcinoma (n = 7); and treated with PORT with non-specific aRTD (n = 118) and treated with PORT with Artd > 60 Gy (n = 14). Stratification log-rank teat was performed to compare the OS between patients treated with/without PORT in different pathological stages and identify at which stage patients would benefit from PORT. Among all 3591 patients, 2765 patients with local advanced T3–4N0 or T1–4N1–3 lesions, among whom PORT group could achieve superior OS compared with non-PORT group and for whom specific information on local-regional disease status was available, were enrolled in a subsequent radiation dose-effect analysis. All the patients in the current study were treated with R0 resection; those who were treated with R1/R2 resection were excluded from the current study. The baseline imaging for the patients included endoscopy with biopsy, barium esophagography, chest/abdominal CT, and cervical ultrasound/CT. For financial reasons, PET-CT was not part of the standard work-up in China between 1993–2012. Patients were followed up until death or April 2019. The median follow-up duration was 65.4 months; disease- and treatment-related characteristics were recorded.

### 2.2. Treatment

Sweet esophagectomy (1697 cases, 47.25%), Ivor–Lewis esophagectomy (104 cases, 2.90%), and McKeown esophagectomy (1790 cases, 49.85%) were performed. Lymphadenectomy in left and right paracardial, subcarinal, left and right bronchial, lower posterior mediastinum, pulmonary ligament, and paraesophageal regions were performed in all patients. Furthermore, paratracheal and left and right recurrent laryngeal nerve lymphadenectomy were performed in patients treated with right thoracotomy. Cervical lymphadenectomy was systematically performed in the McKeown procedure. For patients with pathological T2-4 or N1-3 lesions or T1N0 lesion with risk factors such as LVSI, adjuvant chemotherapy was preferable. Adjuvant chemotherapy could be waived in those who are more likely to discontinue the adjuvant chemotherapy or radiotherapy due to advanced age, complications, large planning target volume, or concerns about the treatment-related toxicities, and preference for relative moderate treatment modality.

Among the 3591 patients treated with radical esophagectomy, 977 received adjuvant therapy subsequently, with 157 patients having received both adjuvant radiotherapy and chemotherapy (106 patients received concurrent chemoradiotherapy and 51 patients received subsequent chemoradiotherapy), 757 patients received adjuvant radiotherapy only, and 63 patients received adjuvant chemotherapy only. The aRTD was converted to the equivalent dose divided in 2 Gy per fraction (EQD2). The EQD2 was calculated based on the linear-quadratic formulation: EQD2 = D × (d + α/β)/(2 Gy + α/β) [32]. The total postoperative radiation dose was described as D, and the radiation dose per fraction was described as d in the formulation. The dose at which the linear α and quadratic β components of cell killing are equal was described as α/β. For ESSC, the α/β value could be considered as 10. For those who did not receive PORT, the aRTD were defined as 0 Gy in the subsequent dose-effect analysis.

### 2.3. Statistical Analyses

OS, DFS, LRFS and distant metastasis (DM) times were calculated from the date of surgery to the date of death, disease recurrence (recurrence in the tumor bed, anastomotic orifice, regional lymph nodes, or metastasis in distant lymph nodes or distant organs) or death, local-regional failure (recurrence in the tumor bed, anastomotic orifice or regional lymph nodes), metastasis in distant lymph nodes or distant organs, or the last follow-up date. The Kaplan–Meier method was performed to estimate survival probabilities, and the log-rank test was used for statistical comparison. The Cox proportional hazard regression model was used to identify independent prognostic factors and evaluate the effects of aRTD in patients treated with different surgical or adjuvant therapy procedures. The proportional hazards assumption was checked with Schoenfeld’s global test before establishing the Cox regression model. All statistical tests were two-sided; *p* < 0.05 was considered to indicate statistical significance.

The non-linear relationships between the continuous aRTD and the hazard ratios (HRs) of OS, DFS, LRFS, DM, or treatment-related morbidity (TRM); and the odds ratio (OR) of anastomotic leak or stenosis were assessed by the Cox proportional hazard regression model or logistics regression model using the restricted cubic spline (RCS) method. The relationships between LRFS and OS or DFS were assessed by the linear regression analysis (LRA). The correlation coefficient (r-value) of LRA, ranging from −1 to 1, was used to measure the linear association. The values −1, 0, and 1 indicate a perfect negative linear correlation, no linear correlation, and a perfect positive linear correlation, respectively. The further r is from 0, the stronger the relationship between the two variables. The correlation is considered strong if the absolute r value is greater than 0.75. All statistical calculations were performed with the R software, version 3.6.2 (R Foundation for Statistical Computing, Vienna, Austria).

## 3. Results

### 3.1. Patient Characteristics

In the current study, 3591 patients with pathologically confirmed ESCC who were treated with radical esophagectomy were enrolled (Supplementary Figure S1). The majority of the patients (3196 cases, accounting for 89.00% of all patients) were aged <70 years. Overall, 5.32%, 24.95%, 18.43%, 5.65%, 37.68%, and 7.96% of the patients were diagnosed with 8th AJCC pathologic stage IB, IIA, IIB, IIIA, IIIB, and IVA, respectively. The median aRTD of patients treated with PORT was 54 Gy. There were 22, 38, 450, and 404 patients treated with aRTD of 1–39 Gy, 40–49 Gy, 50–54 Gy, and 55–60 Gy, respectively. With regard to the 60 patients who were treated with aRTD less than 50Gy, 14 and 20 patients received only 1–39 Gy and 40–49 Gy due to less tolerable to PORT, whereas 8 and 18 patients received 1–39 Gy and 40–49 Gy due to the concern of treatment-related toxicities. Patients’ disease- and treatment-related characteristics are listed in Table 1.

### 3.2. Identification of the Patients Who Potentially Benefit from PORT

**Figure 1:**
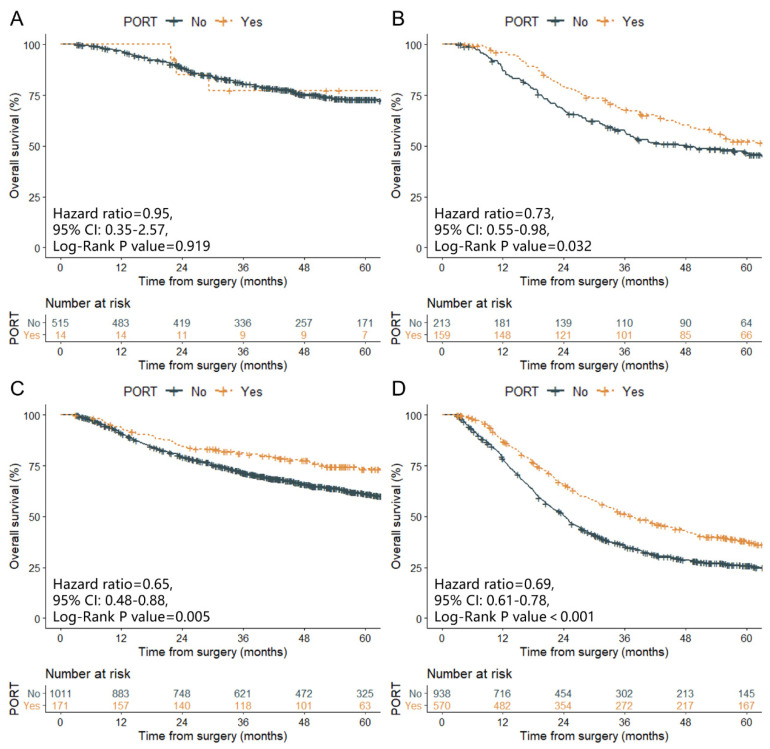
Overall survival of patients with pathologic stage of T1–2N0 (**A**), T1–2N1–3 (**B**), T3–4N0 (**C**), and T3–4N1–3 (**D**).

The multivariable Cox regression model showed that the following parameters were independent prognostic factors of OS: age, sex, thoracotomy procedure, tumor location, tumor length, tumor differentiation, lymph-vascular space invasion (LVSI), T and N stage, postoperative chemotherapy (POCT) modality, and aRTD (Supplementary Figure S2). Regarding the 529 patients diagnosed with T1–2N0 lesions after radical esophagectomy, the OS of the PORT group was similar to that of the non-PORT group (Figure 1A, 5-year OS, 77.38% vs. 72.91%, *p* = 0.919). For the 372, 1182, and 1508 patients diagnosed with T1–2N1–3, T3–4N0, and T3–4N1–3 lesions, respectively, those who received PORT after radical esophagectomy showed superior OS than those who did not (5-year OS, 52.35% vs. 46.60%, *p* = 0.032; 73.41% vs. 61.19%, *p* = 0.005; and 38.30% vs. 25.97%, *p* < 0.001, respectively; Figure 1B–D).

### 3.3. Relationship between Radiation Dose and Survival/Treatment Toxicities

**Figure 2:**
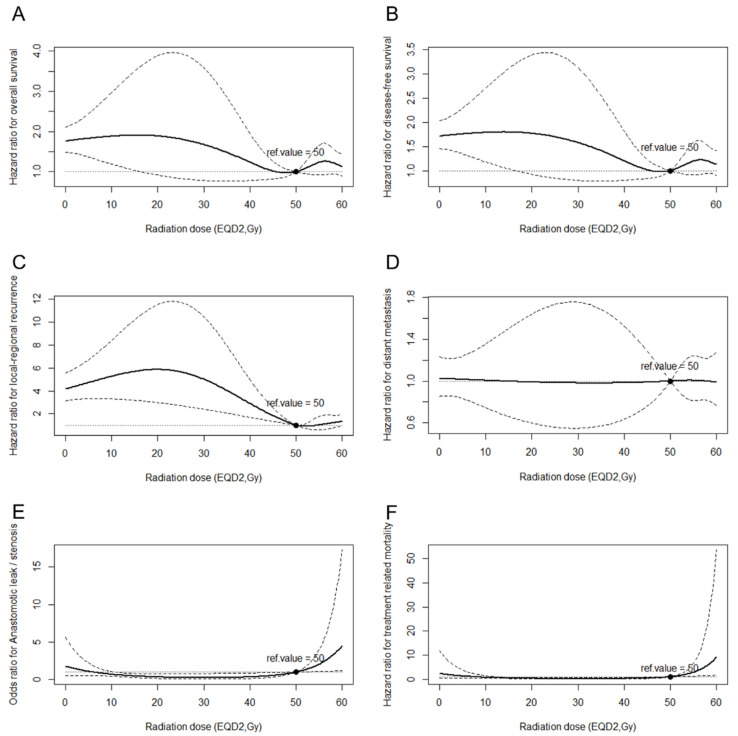
Relationship between adjuvant radiation dose and OS (**A**), DFS (**B**), LRFS (**C**), DM (**D**), anastomotic stenosis/leak (**E**), and TRM (**F**) as analyzed using the restricted cubic spline regression model for 2765 patients with T1-2N1-3 or T3-4N0–3 lesions and available for the analysis of the effect of aRTD.

After adjusting for the general condition factors including age and sex, and disease-related factors including tumor location, tumor length, tumor differentiation, LVSI, and T and N stages, and adjuvant therapy-related factors including POCT modality and radiation technique to reduce the influence of these confounders, the RCS method in the Cox proportional hazard regression model or logistic regression model was applied. The analysis revealed non-linear relationships between continuous aRTD and OS, DFS, LRFS, DM, TRM, and the incidence of anastomotic leak/stenosis in 2765 patients with T3–4N0 or T1-4N1–3 lesions and specific local-regional status information. When the aRTD escalated from 0 Gy (non-PORT group), the HRs of disease recurrence and death decreased until the aRTD approached 50 Gy, and then increased subsequently (Figure 2A,B). The HR of local-regional recurrence decreased as the aRTD escalated and then remained steady when the aRTD exceeded 50 Gy (Figure 2C), whereas the HR of DM remained stable (Figure 2D). In terms of treatment-related toxicities, the trend of OR of anastomotic leak/stenosis was at a steady low level until the aRTD approached 50 Gy, after which there was a sharp increase (Figure 2E). Likewise, the HR of TRM was steady until the aRTD approached 50 Gy; it increased thereafter (Figure 2F).

### 3.4. Effect of the aRTD on LRFS and the Correlation between LRFS and OS/DFS

**Figure 3:**
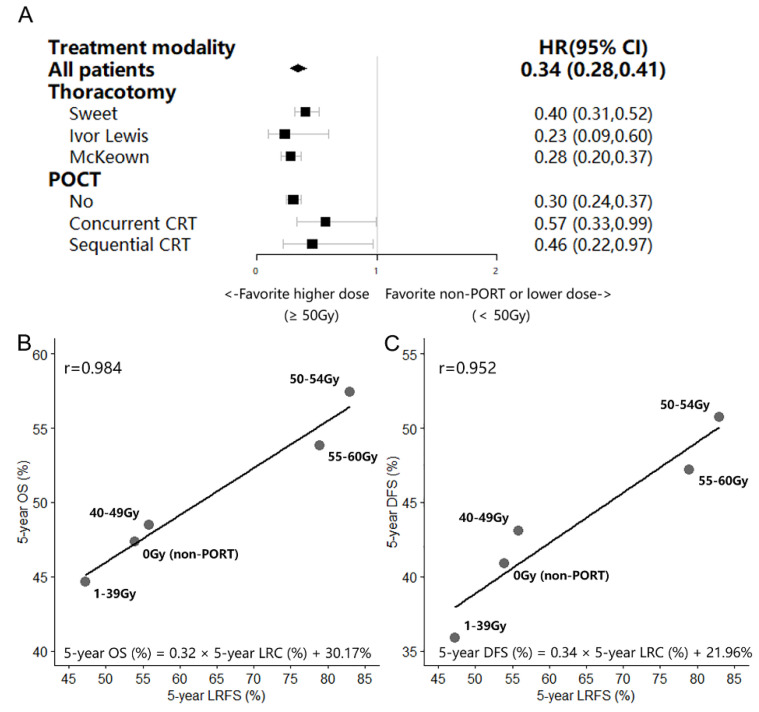
Stratified comparison of LRFS between patients treated with adjuvant radiation dose <50 Gy and ≥50 Gy (**A**) and linear regression analysis of LRFS and OS, DFS according to the adjuvant radiation dose (**B**,**C**) for 2765 patients with T1-2N1-3 or T3-4N0–3 lesions and available for the analysis of the effect of aRTD.

Regarding the patients with T3–4N0 or T1-4N1–3 lesions after radical esophagectomy, the Cox proportional hazard regression model indicated a 66% (range: 59–82%) decline in those treated with a higher Artd (EQD2 ≥ 50 Gy) comparing with that in those treated with a lower Artd (EQD2 < 50 Gy). In terms of patients treated with different surgical or adjuvant therapy procedures (Figure 3A), LRFS benefit was achieved in those who received a high Artd.

The 5-year OS, DFS, and LRFS of patients treated with several Artd levels (0 Gy, 1–39 Gy, 40–49 Gy, 50–54 Gy, and 55–60 Gy) ranged from 44.64% to 57.44%, 35.90% to 50.74%, and 47.20% to 83.03%, respectively. Patients who received an Artd (EQD2) in the range of 50–54 Gy tended to show better OS, DFS, and LRFS (Table 2). LRA revealed strong correlations between improved LRFS and increased OS, DFS (r = 0.984 and r = 0.952, respectively). An absolute advancement of 1% in the LRFS translated into improvements of 0.32% and 0.34% in the OS and DFS, respectively (Figure 3B,C).

## 4. Discussion

Based on the data of the large real-world cohort in 2 cancer centers in China, the current multicenter retrospective study explored the trends of risk of death, disease recurrence, local-regional recurrence, distant metastasis, treatment-related toxicities, and treatment-related mortality as aRTD continuously escalated. Moreover, the correlation between LRFS and OS/DFS were quantitatively evaluated. The log-rank test showed that after upfront surgical resection, patients with pathological T3–4N0 or N1–3 ESCC lesions could be the benefit carriers of PORT. Furthermore, the restricted cubic spline model revealed that 50Gy could be the satisfied adjuvant radiation dose threshold that can well balance PORT-related survival benefit and toxicities. Finally, in further linear regression analysis, we found that the LRFS enhancement from PORT with appropriate aRTD could lead to improvement in OS and PFS.

Over 50% of patients with esophageal cancer receive surgical resection as primary management in clinical practice [4,5]. For patients treated with upfront surgical resection, local-regional recurrence accounted for 55.6–84.5% of the overall disease recurrence [6,7,8,9,10]. Moreover, the incidence of local-regional recurrence was still similar between patients with or without adjuvant chemotherapy followed by surgical resection [33]. PORT was proved to be an essential treatment approach for reducing the risk of local-regional recurrence [13,14,15,16,17,18,19,20,21]. However, there were few studies in the literature investigating the extent to which LRFS influences survival outcomes and how PORT-related gains of LRFS translate into survival improvement in real-world clinical practice [34]. In the current study, the linear regression models of the survival data from two cancer centers revealed a correlation between LRFS and survival outcomes. Improved LRFS was strongly correlated to OS and DFS increase (r = 0.984 and r = 0.952, respectively). An absolute enhancement of 1% in the LRFS resulting from PORT could translate into improvements of 0.32% and 0.34% in OS and DFS, respectively. It was rational to assume that with the aRTD escalation, PORT-related LRFS improvement could lead to subsequently increased DFS and OS. However, the incidence of treatment-related toxicities, which could result in low QoL or treatment-related mortality, would increase along with the aRTD escalation. Hence, it was supposed that there was an aRTD threshold at which the greatest extent of LRFS enhancement-related survival improvement could be attained along with tolerable treatment-related toxicities. 

In the current study, the RCS method in the Cox proportional hazard regression models and the logistics regression model were applied to evaluate the relationship between continuous aRTD and the risk of treatment-related toxicities, disease recurrence, or death. As the aRTD escalated from 0 to 50 Gy, the risk of local-regional recurrence continuously decreased, with the risk of anastomotic stenosis/leak and treatment-related mortality remaining at a steady low level. In this phase, the gain of PORT-related LRFS enhancement suppressed the toxicity-related impairment in OS and DFS, and the risk of death was continuously decreased. Once the aRTD exceeded 50 Gy, the risks of anastomotic stenosis/leak and treatment-related mortality increased sharply, without a further decrease in the risk of local-regional recurrence. In this phase, the loss of survival benefits resulting from treatment-related toxicity and the negative effects of the accompanying discontinuous treatment overshadowed the gain of PORT-related LRFS enhancement. The risk of death thereby increased. The aRTD of 50 Gy was deemed to balance the toxicity-related survival impairment and survival gain associated with the absence of disease recurrence to the greatest extent. Similarly, the retrospective study established by Moon [28] et al. showed that patients who were treated with aRTD of 50 Gy or higher (median 54 Gy) could achieve superior DFS and local-regional control compared with those who were treated with aRTD lower than 50 Gy (median 45 Gy), and the risk of grade 3 or higher complications were similarly low in the 2 aRTD groups. In the neoadjuvant and definitive setting, the recommendation of radiation dose was controversial; following the protocol of CROSS [1] and NEOCRTEC5010 [2] trial, the recommended neoadjuvant radiation dose (nRTD) was 41.4 or 40 Gy. The retrospective analysis of the National Cancer Database (NCDB) showed that patients who were treated with nRTD more than 50.4 Gy could encounter higher 30-day mortality without a higher pathological complete response (pCR) rate comparing to those who were treated with nRTD ranging 40–50.4 Gy. Furthermore, further analysis between the patients treated with nRTD of 50.4 Gy and 45 Gy showed that nRTD of 50.4 Gy could achieve a higher pCR rate without increased treatment-related mortality [35]. In terms of the definitive radiation dose (dRTD), based on the result of RTOG 8501 [36] and RTOG 9405 [37] in conventional RT era, the recommended dRTD was 50–50.4 Gy. In several previous phase 1/2 clinical trials [38,39,40,41], chemoradiotherapy with simultaneous integrated boost (SIB) of dRTD over 60Gy to the primary tumor and metastatic lymph nodes was well tolerated with acceptable toxicities; moreover, it could attain superior local-regional control and OS compared with the contemporaneous institutional cohort treated with dRTD of 50.4 Gy. Nevertheless, the IMRT-based ARTDECO study [42] failed to reveal the survival benefit of dRTD over 60 Gy. As the patients with unresectable lesions were heterogeneous in radiation sensitivity, only 33.3% of the patients attained clinical complete response (cCR) and achieved long term survival after receiving dRTD of 50 Gy. In terms of the patients who could not attain cCR after receiving dRTD of 50 Gy (accounted for 66.7% of all patients), over 50% of them encountered local relapse with a median subsequent survival time of 13.3 months [43]. For those who failed to attain cCR after receiving dRTD of 50 Gy, the radiation dose of 50 Gy might be insufficient given the high local relapse rate and poor prognosis. Therefore, patients who could not attain cCR with dRTD of 50 Gy might be the potential benefit carriers of SIB of dRTD of 60 Gy. Further prospective stratification randomized controlled trials based on the radiation sensitivity are warranted to evaluate the effect of dRTD of 60 Gy. 

When PORT with appropriate aRTD was applied after upfront surgical resection, the patients with local advanced T3-4N0 or T1-4N1-3 lesions could achieve lower local-regional recurrence rate and prolongation of survival. However, as the continuous dose-response smooth HR curve in the current study showed the HR of DM remained stable with aRTD escalation, DM might account for a larger proportion of the failure pattern in PORT-treated patients and become the major concern in the adjustment of intervention strategies. The addition of concurrent or sequential chemotherapy might be one of the potential approaches to consolidate the LRFS enhancement-related survival benefit. PORT with concurrent chemotherapy was demonstrated to result in tolerable toxicities and potential survival benefits comparing with PORT alone [15,25]. Nevertheless, further prospective trials with larger samples and longer follow-ups are warranted. Furthermore, the recently established CheckMate 577 trial [44] revealed the addition of adjuvant nivolumab for patients who failed to achieve a pathological complete response after neoadjuvant chemoradiotherapy, and subsequent surgical resection could afford superior DFS. As the prognosis of patients receiving radical surgical resection was analogously heterogeneous depending on differences in tumor burden, the addition of immunotherapy to PORT might be another potential approach for reducing DM risk.

Despite receiving R0 resection, patients pathologically diagnosed with T3–4N0 or T1-4N1–3 lesions were at a higher risk of disease recurrence comparing with that in the patients with T1–2N0 lesions [7]. Local-regional recurrence was the failure pattern in the majority of patients diagnosed with local advanced stage disease after receiving radical surgical resection as primary treatment. In addition, 23.0%–56.5% of patients with local advanced lesions were at risk of local-regional recurrence [6,7,8,9,10] with a limited survival of 3–8 months thereafter [10,11,12]. The concern of consolidating adjuvant local treatment remained a key issue for patients at high risk of local-regional recurrence to achieve a more favorable prognosis. In the current study, multivariable analysis showed that the pathologic T and N stage were the independent prognostic factors affecting OS. Subsequently, stratification analysis showed that PORT could result in an increase of 5.75%, 12.22%, and 12.33% in OS for patients with T1–2N1–3, T3–4N0, and T3–4N1–3 lesions, respectively. A similar conclusion was found in a prospective study [15]. Although the calculated sample size was not achieved in the prospective trial, OS and DFS improvements because of PORT or POCRT were still observed in patients with lesions of stage IIB or higher. Likewise, retrospective studies of the Surveillance Epidemiology and End Results (SEER) [19] and National Cancer Data Base (NCDB) [20] database demonstrated that PORT could result in improved OS for patients with T3–4N0M0 and T1–4N1M0 lesions or node-positive disease. Patients with a local advanced tumor burden are at a higher risk of death; however, they are also the potential beneficiaries of PORT.

There is robust evidence supporting the importance of PORT in prolonging local-regional recurrence or attaining superior DFS or OS in selected patients [13,14,15,16,17,18,19,20,21]. However, concerns about severe treatment-related toxicities, such as anastomotic stenosis or anastomotic leak, which might lead to a low QoL due to persistent dysphagia or treatment-related mortality, limit the application of PORT in clinical practice. Several prospective randomized trials conducted in the last century failed to reveal the effectiveness of PORT in improving the OS of patients treated with upfront surgery [45,46,47]. Although the incidence of local-regional recurrence in patients receiving PORT was significantly lower than that in those who received surgery alone, the high incidence of severe treatment-related adverse events resulting from old radiation techniques and insufficient extensive target volumes or radiation dose prescription could offset the survival benefit obtained from PORT-related LRFS improvement. The application of modern IMRT techniques in PORT was shown to result in acceptable treatment-related toxicities in several prospective trials [14,15]. Hence, the role of PORT must be re-evaluated. In the current study, stratification analysis showed that a higher aRTD was correlated with LRFS enhancement in patients receiving different surgical or adjuvant therapy procedures, which was consistent with previous findings [28]. Additionally, the continuous dose-response smooth HR curve in the current study revealed the risks of anastomotic stenosis/leak and treatment-related mortality were low at an aRTD ≤50 Gy. The application of PORT with an adequate radiation dose could contribute to LRFS benefits in patients with local advanced lesions.

The main limitation of our study was its retrospective design, which may have introduced selection bias into the results and conclusion. Considering that a large population with long-term follow-up from two cancer centers located in a high-risk region for esophageal carcinoma in China was enrolled, until confirmation is obtained in well-designed randomized controlled trials, the findings of the current study could serve as evidence for designing additional prospective stratified randomized controlled trials. In addition, because of the limited number of patients who received both PORT and POCT in this study, although the effect of postoperative chemoradiotherapy (concurrent or sequential) on survival outcomes were analyzed through multivariable and stratified univariable Cox regression, the effect of postoperative chemoradiotherapy on treatment-related toxicities was not analyzed. Furthermore, due to the retrospective nature of the current study, the information of PTV volumes, which was one of the important factors of radiation toxicities, was unavailable for the patients who were treated with PORT and thus, they were not enrolled in the analysis, which may limit our conclusions on toxicity. Therefore, further prospective trials or retrospective studies based on real-world data with a large population are warranted.

## 5. Conclusions

Patients pathologically diagnosed with local advanced T3–4N0 or T1-4N1–3 lesions after upfront radical surgical resection are potential beneficiaries of PORT. An aRTD of 50 Gy could yield favorable LRFS with acceptable toxicities for patients with T3–4N0 or T1-4N1–3 lesions after upfront radical surgical resection. The LRFS gains obtained from PORT could translate into considerable survival benefits. Clinicians should be aware that PORT has the potential to improve unfavorable LRFS and survival outcomes in ESCC patients treated with upfront surgery. The findings of the current study could serve as evidence for delivering appropriate aRTD and designing additional prospective stratified randomized controlled trials.

## Figures and Tables

**Table 1:** Baseline clinicopathological characteristics of the included patients.

Characteristics	No. (%) (All 3591 Cases)
Age	
<70 years	3196 (89.00%)
≥70 years	395 (11.00%)
Median (IQR) [years]	58 (52–65)
Sex	
Male	2777 (77.33%)
Female	814 (22.67%)
Thoracotomy	
Sweet	1697 (47.25%)
Ivor–Lewis	104 (2.90%)
McKeown	1790 (49.85%)
Location	
Upper third	338 (9.41%)
Middle third	2174 (60.54%)
Lower third	1079 (30.05%)
Length	
≤5 cm	2121 (59.06%)
>5 cm	1470 (40.94%)
Median (IQR) [cm]	5 (4–6)
Differentiation	
Well	676 (18.82%)
Moderate	2062 (57.42%)
Poorly	853 (23.75%)
LVSI	
no	3108 (86.55%)
yes	483 (13.45%)
T stage (AJCC 8th)	
T1b	191 (5.32%)
T2	710 (19.77%)
T3	2492 (69.40%)
T4a	198 (5.51%)
N stage (AJCC 8th)	
N0	1711 (47.65%)
N1	1035 (28.82%)
N2	622 (17.32%)
N3	223 (6.21%)
TNM stage (AJCC 8th)	
IB	191 (5.32%)
IIA	896 (24.95%)
IIB	662 (18.43%)
IIIA	203 (5.65%)
IIIB	1353 (37.68%)
IVA	286 (7.96%)
POCT	
No	3371 (93.87%)
POCT without RT	63 (1.76%)
Concurrent CRT	106 (2.95%)
Subsequent CRT	51 (1.42%)
PORT	
No	2677 (74.55%)
Yes	914 (25.45%)
Radiation technique	
Conventional RT (2D-RT)	191 (20.90%)
3D-CRT	41 (4.48%)
IMRT	678 (74.62%)
Radiation dose (EQD2)	
1–39Gy	22 (2.41%)
40–49Gy	38 (4.16%)
50–54Gy	450 (49.23%)
55–60Gy	404 (44.20%)
Median (IQR) [Gy]	54 (50–60)

LVSI: lymph-vascular space invasion, POCT: postoperative chemotherapy, CRT: chmoradiotherapy, PORT: postoperative radiotherapy, 2D-RT: 2 dimension radiotherapy, 3D-CRT: 3 dimension radiotherapy, IMRT: intensity-modulated radiotherapy, and EQD2: equivalent dose in 2 Gy per fraction.

**Table 2:** OS, DFS, and LRFS of the radiation dose groups for 2765 patients with T1-2N1-3 or T3-4N0–3 lesions and available for the analysis of the effect of aRTD.

Radiation Dose (EQD2)	No.	5-Year OS (%)	Log-Rank *p*-Value	5-Year DFS (%)	Log-Rank *p*-Value	5-Year LRFS (%)	Log-Rank *p*-Value
0 Gy (non-PORT)	1987	47.37	0.001	40.88	0.001	53.89	<0.001
1–39 Gy	16	44.64		35.90		47.20	
40–49 Gy	37	48.50		43.07		55.85	
50–54 Gy	369	57.44		50.74		83.03	
55–60 Gy	356	53.80		47.17		78.90	

EQD2: equivalent dose in 2 Gy per fraction, OS: overall survival, DFS: disease-free survival, and LRFS: local-regional recurrence free survival.

## Data Availability

The data underlying this article will be shared on reasonable request to the corresponding author.

## Supplementary Materials

The following supporting information can be downloaded at: https://www.mdpi.com/article/10.3390/cancers14235879/s1, Figure S1: CONSORT diagram of patient selection; Figure S2: Forest plots of Cox proportional hazard regression multivariable analysis of overall survival.
