# Supplementary material for: Effect of Adjuvant Radiation Dose on Survival in Patients with Esophageal Squamous Cell Carcinoma
Source: Cancers (Basel). 2022 Nov 29;14(23):5879. doi: 10.3390/cancers14235879 (PMC9736548; doi:10.3390/cancers14235879)
Supplement: Supplementary file 1 [file cancers-14-05879-s001.zip › cancers-1996705-supplementary.pdf]

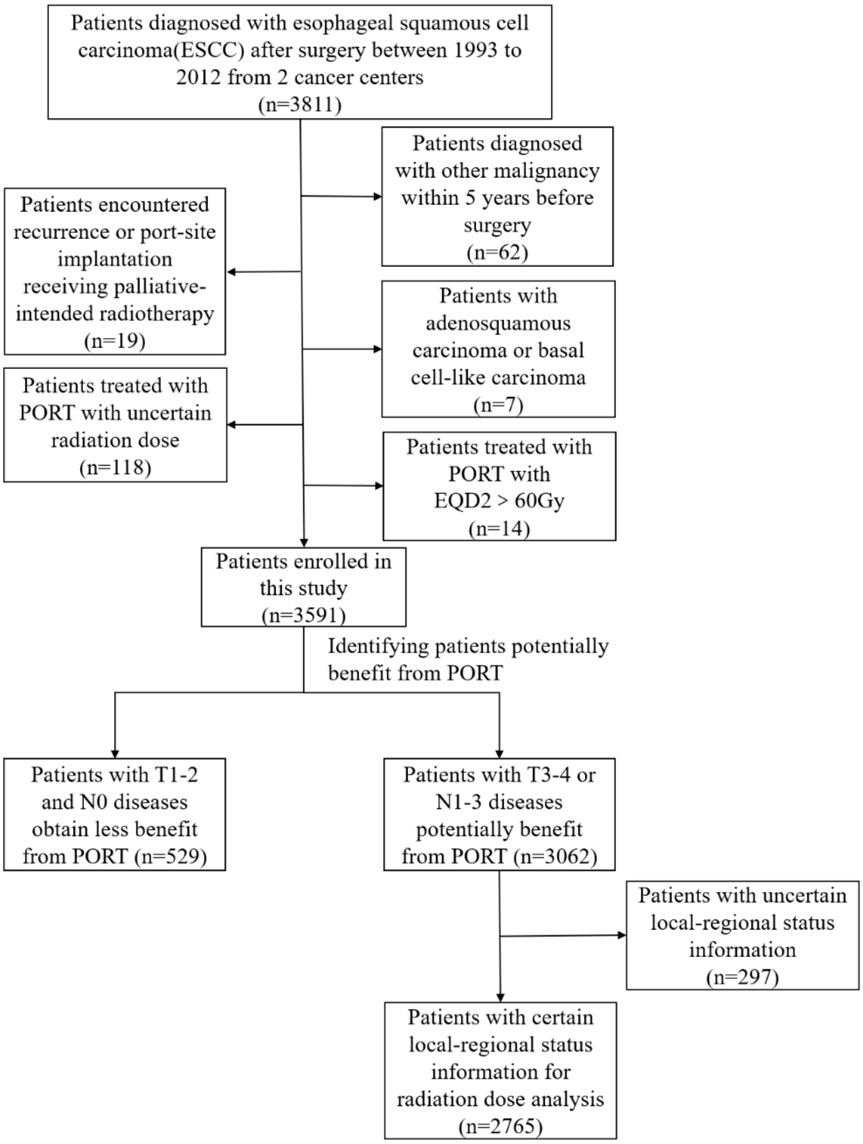

Supplementary Figure S1. CONSORT diagram of patient selection.

Characteristics	HR(95% CI)	p value
Age (> 70 years vs ≤70 years)	1.34 (1.17,1.55)	< 0.001
Sex (female vs male)	0.78 (0.69,0.88)	< 0.001
Thoracotomy procedure		0.002
Ivor Lewis vs. Sweet	0.95 (0.73,1.26)	0.735
McKeown vs. Sweet	0.82 (0.73,0.92)	0.001
Location		< 0.001
middle third vs. upper third	0.86 (0.73,1.01)	0.070
lower third vs. upper third	0.67 (0.55,0.82)	< 0.001
Length (> 5cm vs. ≤5cm)	1.10 (1.00,1.21)	0.049
Differentiation		0.045
moderate vs. well	1.07 (0.94,1.22)	0.317
poorly vs. well	1.19 (1.03,1.39)	0.020
LVSI (yes vs. no)	1.27 (1.12,1.45)	< 0.001
T stage		< 0.001
T2 vs. T1b	1.46 (1.10,1.95)	0.009
T3 vs. T1b	1.98 (1.51,2.58)	< 0.001
T4a vs. T1b	2.44 (1.78,3.36)	< 0.001
N stage		< 0.001
N1 vs. N0	2.17 (1.92,2.44)	< 0.001
N2 vs. N0	3.28 (2.86,3.76)	< 0.001
N3 vs. N0	4.96 (4.15,5.93)	< 0.001
Chemotherapy modality		0.025
POCT without RT vs. Non-POCT	0.71 (0.50,1.01)	0.057
Concurrent CRT vs. Non-POCT	0.74 (0.56,0.99)	0.040
Subsequent CRT vs. Non-POCT	0.74 (0.51,1.07)	0.109
Radiation technique		0.890
3D-CRT vs. conventional RT	0.98 (0.61,1.57)	0.923
IMRT vs. conventional RT	0.94 (0.74,1.21)	0.645
Radiation dose (EQD2, Gy)		< 0.001
1-39Gy vs. 0Gy (non-PORT)	0.99 (0.53,1.86)	0.979
41-49Gy vs. 0Gy (non-PORT)	0.83 (0.53,1.29)	0.408
50-54Gy vs. 0Gy (non-PORT)	0.68 (0.59,0.79)	< 0.001
55-60Gy vs. 0Gy (non-PORT)	0.69 (0.58,0.80)	< 0.001

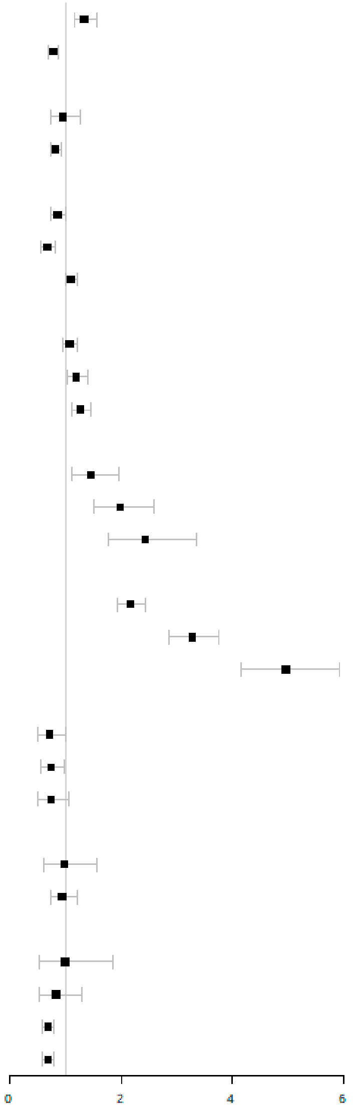

Supplementary Figure S2. Forest plots of Cox proportional hazard regression multivariable analysis of overall survival.
